# Formation Regularities of Plasmonic Silver Nanostructures on Porous Silicon for Effective Surface-Enhanced Raman Scattering

**DOI:** 10.1186/s11671-016-1473-y

**Published:** 2016-05-21

**Authors:** Hanna V. Bandarenka, Kseniya V. Girel, Vitaly P. Bondarenko, Inna A. Khodasevich, Andrei Yu. Panarin, Sergei N. Terekhov

**Affiliations:** Laboratory of Materials and Structures of Nanoelectronics, Belarusian State University of Informatics and Radioelectronics, 6 Brovka st., Minsk, 220013 Belarus; B.I. Stepanov Institute of Physics of National Academy of Sciences of Belarus, 68 Nezalezhnasti av., Minsk, 220072 Belarus

**Keywords:** Porous silicon, Silver nanoparticles, Immersion deposition, Surface-enhanced Raman scattering, Detection limit

## Abstract

Plasmonic nanostructures demonstrating an activity in the surface-enhanced Raman scattering (SERS) spectroscopy have been fabricated by an immersion deposition of silver nanoparticles from silver salt solution on mesoporous silicon (meso-PS). The SERS signal intensity has been found to follow the periodical repacking of the silver nanoparticles, which grow according to the Volmer–Weber mechanism. The ratio of silver salt concentration and immersion time substantially manages the SERS intensity. It has been established that optimal conditions of nanostructured silver layers formation for a maximal Raman enhancement can be chosen taking into account a special parameter called effective time: a product of the silver salt concentration on the immersion deposition time. The detection limit for porphyrin molecules CuTMPyP4 adsorbed on the silvered PS has been evaluated as 10^−11^ M.

## Background

Surface-enhanced Raman scattering (SERS) spectroscopy is widely recognized as a label-free high-sensitive method for applications in analytical chemistry [1, 2], biomedicine [3–5], and various sensor devices [6–9]. SERS is observed for analyte molecules adsorbed on the noble metal surfaces with nanoscale roughness, so-called SERS-active substrates [10, 11]. An enormous level of Raman signal in SERS is mainly attributed to the strong enhancement of the local electromagnetic field near the metallic nanostructures or junctions between them [12, 13], resulted from the excitation of surface plasmon resonance (SPR)—the coherent oscillations of electrons at metal-dielectric interfaces [14, 15].

A fabrication of novel effective and inexpensive plasmonic nanostructures with well-defined nanoscale geometry, which demonstrate strong, reproducible, and stable Raman signal, is an important current challenge to make SERS spectroscopy a routine practical method for a wider range of applications. Accordingly, a number of different methods have been developed for the formation of plasmonic materials suitable for SERS, as might be evidenced from the recent reviews [16, 17].

Metallic nanoparticles (NPs) of different size and shape synthesized through ordinary wet chemistry and immobilized on solid substrates by various techniques have been thoroughly studied and extensively used in SERS experiments [10, 18]. There are many ways of immobilization of the metallic NPs on the solid substrates including chemical attachment with the use of bifunctional molecules, self-assembly based on electrostatic attraction, using capillary force as a driving mechanism for deposition, and chemical and photochemical growth. However, the abovementioned methods often cause dispersion and aggregation of the metallic NPs making difficult to provide enough reproducibility of the characteristics of SERS-active substrates in practice. To overcome the aforementioned limit, modern nanofabrication approaches such as electron-beam and nanosphere lithography, Langmuir–Blodgett technique and self-ordering method have been proposed [19–21]. However, these technologies are rather high cost and require substantial experience of an operator.

In terms of simplicity and cost-effectiveness, a lithography-free approach based on the using porous templates is one of the most promising ways to fabricate plasmonic nanostructures with controlled geometry, long-range ordering, and well-defined size and shape. Recently, it has been shown that metalized porous silicon (PS) demonstrates promising features for effective SERS. PS is known for its exceptional properties such as the extremely high effective surface area, large adsorption capacity, photo- and electroluminescence, and biocompatibility [22, 23]. Metallic coatings can be formed on the PS template via various techniques: thermal decomposition of metal salt in an oxidized PS [24, 25]; metal-assisted wet etching and sputtering processes [26]; physical vapor deposition [27]; inject printing synthesis [28]; and immersion plating of metals within the pores of PS [29–31]. The most significant advantage of the PS template is a possibility to vary and precisely control structural parameters of the porous layers, thus affecting the morphology of such plasmonic metal nanostructures.

The immersion plating occupies a special place among the other metal deposition techniques as this method does not require vacuum equipment, energy supply or special reducing agents: it is carried out due to redox reactions between atoms of a substrate and metal ions. In case of Si-based substrates, solutions containing ions of metals with positive redox potential (Cu and noble metals) have to be used to provide the immersion deposition [32]. By nowadays, several reports have described immersion deposition of gold [33], copper [34], and silver [35–40] on PS at different conditions to form SERS-active substrates*.* Silver deposits have been mainly used to form SERS-active substrates based on PS due to the strongest plasmonic properties of this metal [41]. A dependence of the Ag nanostructure morphology and SERS activity on various PS parameters (thickness, porosity, and pore diameter) and Ag deposition regimes (an immersion time, a temperature, and a concentration of the solution reagents) have been investigated [38].

In the issue of aforementioned works, the properties of Ag nanostructures fabricating at the same immersion solution temperature on the identical PS samples have been found to depend on the following main factors: (i) the concentration of Ag ions and (ii) the Ag deposition duration. Nevertheless, these two factors have been hardly combined for different cases of the SERS-active substrates formation.

Giorgis’ group has carefully studied a morphology of Ag samples synthesized by the PS immersion in a 1–10 mM AgNO_3_ aqueous solution for 0.5–10 min at a temperature of 5–50 °C [30, 37]. The strongest Raman enhancement has been observed for the case of an excitation wavelength matching with the SPR band of the Ag/PS substrates. Recently, it has been reported detection of crystal violet with Ag nanostructures formed by the PS immersion in a 10 mM AgNO_3_ aqueous solution for a period ranging from 15 to 120 s [36]. The most intensive SERS signal has been typical for the substrate prepared by the 30s immersion plating. Zeiri et al. [35] have studied the silver immersion deposition on PS in the AgNO_3_ solutions, which contain different concentration of Ag^+^ ions (from 1 to 500 mM). The deposition time was 30 min. The largest SERS activity was reached for the substrate formed with the 50 mM Ag^+^ solution. We have investigated SERS-active substrates prepared on the PS templates based on *p-* and *n*-type silicon wafers by the immersion deposition for 1 to 30 min in 1, 10, and 100 mM AgNO_3_ solutions [31, 38]. The best SERS results for rhodamine 6G probing molecule have been obtained for the samples formed by the Ag deposition in a 10 mM AgNO_3_ aqueous solution during 10–15 min and 5 min for the PS samples based on *p-* and *n-*type silicon wafers, respectively.

An ultimate goal of the described works was to reveal correlation between the concentration of the Ag salt and the deposition time to find optimal fabrication conditions for the most effective SERS. Obviously, an increase of the Ag^+^ concentration in the solution leads to the Ag deposition fastening. However, the rapid process makes difficult careful control of the morphology of the Ag structures, which can coalesce into continuous film or large aggregates not favorable for SERS activity. On the other hand, a highly diluted Ag salt solution results in a very slow silver deposition. Moreover, H^+^ ions in aqueous solutions can compete with Ag^+^ ions in the oxidation of the silicon atoms. In this case, the PS surface will be covered with silicon oxide before formation of sufficient amount of silver deposit. Therefore, the question arises on the choice of proper conditions of the Ag immersion deposition on PS, i.e., relation between the Ag salt concentration and the immersion duration.

Herein, we report a systematic study of the Ag nanostructures formed by the immersion technique on the PS surface. We used the highly doped *n*^*+*^-type silicon wafers as it allows fabrication of the mesoporous silicon (meso-PS) composed of ordered vertical pores. Such material is a perfect template for the metallic deposit formation with required spatial location. The appropriate Ag nanostructures were produced in three solutions of different AgNO_3_ concentration. The deposition duration was varied in the wide range from a few to 200 min. SERS efficiency of the Ag/PS substrates was evaluated using water-soluble cationic porphyrin CuTMPyP4. Based on the analysis of the regularities of the Ag nanostructures growth and corresponding kinetics of SERS-signal intensity change, we revealed a parameter crucial for the formation of the SERS-active substrates, which can be defined as a product of the Ag salt concentration by the immersion deposition time.

## Methods

Antimony doped 100 mm monocrystalline Si wafers of (100) orientation, and 0.01 Ω cm resistivity were used as an initial substrates. A chemical cleaning of the Si wafers was performed for 10 min in a hot (75 °C) solution of NH_4_OH, H_2_O_2_, and H_2_O mixed in a volume ratio of 1:1:4. The Si wafers rinsing with a deionized water and drying in a centrifuge followed the cleaning. Then the Si wafers were cut into a number of rectangular samples with an area of 9 cm^2^. Some of the samples were used to deposit Ag on the surface of the original monocrystalline Si for a comparison of the immersion process on both the Si and PS templates. Just before the PS formation or the Ag immersion deposition, each Si sample was dipped in a diluted HF (5 %) for 30 s to remove a native oxide. Immediately after the native oxide removal, the Si sample was placed in an electrolytic cell made of polytetrafluoroethylene. An active opening of the cell had a round shape and an area of 3 cm^2^. A highly pure graphite disk was used as a contact electrode to a backside of the samples during the electrochemical treatment. A platinum spiral wire was used as a cathode electrode. The PS layers were formed by an electrochemical anodization of the Si samples in a solution of HF (45 %), H_2_O, and С_3_Н_7_ОН mixed in a 1:3:1 volume ratio according to the procedure described elsewhere [42]. The anodization was performed at a current density of 100 mA/cm^2^ for 85 s. Such anodizing regimes provided fabrication of uniform PS layers of a 5-μm thickness and an average pore diameter of 60 nm at the top of PS layer. The resulting PS sample was kept for 30 min in C_3_H_7_OH to remove the products of the electrochemical process from the pores.

The Ag deposition on the samples of Si and PS was carried out by the immersion plating in 1, 3, and 10 mM AgNO_3_ aqueous solutions with an ethanol additive. The immersion continued for 5–280 min. The Ag-coated samples were thoroughly rinsed with the deionized water and ethanol and then dried in the air.

The SERS activity of the experimental samples was studied using a cationic Cu(II)-tetrakis(4-*N*-methylpyridyl) porphyrin (CuTMpyP4) which was synthesized according to standard procedures by Dr. V.L. Malinovskii (Bogatsky Physico-Chemical Institute, Odessa, Ukraine). The samples for the SERS measurements were prepared by an incubation of the Ag-coated PS in the analyte aqueous solution of various molar concentrations (10^−6^–10^−12^ M) during 2 h. Prior to impregnation with the analyte solution, the Ag-coated substrates were rinsed with a solution of HCl and H_2_O mixed in 1:99 volume ratio. It provided an elimination of contaminants adsorbed on a high surface area of the Ag deposit that results in a strong SERS background.

All abovementioned procedures were carried out at the room temperature. The potentiostat/galvanostat AUTOLAB PGSTAT302n (The Netherlands) was used to conduct electrochemical processes.

Gravimetric method was applied to determine the porosity of PS. Mass measurements were performed with a Sartorius CP225D micro/analytical electronic balance (Germany). The instrumental mass error was 10 μg. The morphology of the samples was studied by the scanning electron microscopy (SEM; Hitachi S-4800, Japan) with 1 nm resolution. The phase composition of the samples was determined by X-ray diffraction (XRD) using CuKα radiation (X-ray wavelength *λ* = 0.15406 nm).

The electronic absorption spectra of the CuTMpyP4 solutions were measured with a Cary 500 Scan spectrophotometer (Varian, USA) in 10 × 10 mm quartz cells. Reflectance spectra of the Ag-coated samples were measured with the spectrophotometer MC 122 (Proscan Special Instruments, Belarus) in the range of 200 to 1000 nm. SERS spectra were recorded with 90° scattering geometry by using Solar TII DM160-MS3504I spectrometer equipped with a CCD detector SPEC10:256E (Roper Scientific, USA) cooled down to 153 K with liquid nitrogen. Excitation at 441.6 nm was provided by He–Cd laser (Liconix, USA). The cylindrical lens was used to stretch out the spot to 5 mm in length. All measurements were made at the room temperature.

## Results and Discussions

### Structure of the Ag-Coated PS Samples

During the process of immersion deposition of silver, meso-PS plays not only a role of the template defining a shape, dimensions, and spatial location of the Ag nanostructures. It also acts as a source of Ag nucleation centers and electrons for the reduction of Ag^+^ ions that allows avoiding the use of special reducing agents and surface activators, which can be incorporated in the Ag deposit, thus resulting in an undesirable noise in SERS spectra. The Ag film formation on the PS surface proceeds in accordance with the Volmer–Weber mechanism [43] and the model proposed by Ogata’s group [44]. PS acts as a mild reducing agent, which can spontaneously reduce metallic ions which have positive reduction potentials with respect to hydrogen. We checked the possibility of Ag film formation on the monocrystalline Si wafer by the immersion deposition. Despite a small amount of the Ag deposit was observed on the Si surface, no SERS activity was revealed for those samples. It is explained as follows: an exposure of the monocrystalline Si in the Ag salt aqueous solutions causes simultaneously with growth of Ag nanoparticles and a continuous layer of SiO_2_. The oxide layer prevents contact of AgNO_3_ with the Si atoms resulting in the limitation retardation of the metal reduction. Below, it will be shown that an extremely developed specific surface area of PS and its open structure provide formation of an enough amount of the nanostructured Ag deposit favorable for the high efficient SERS activity.

**Fig. 1:**
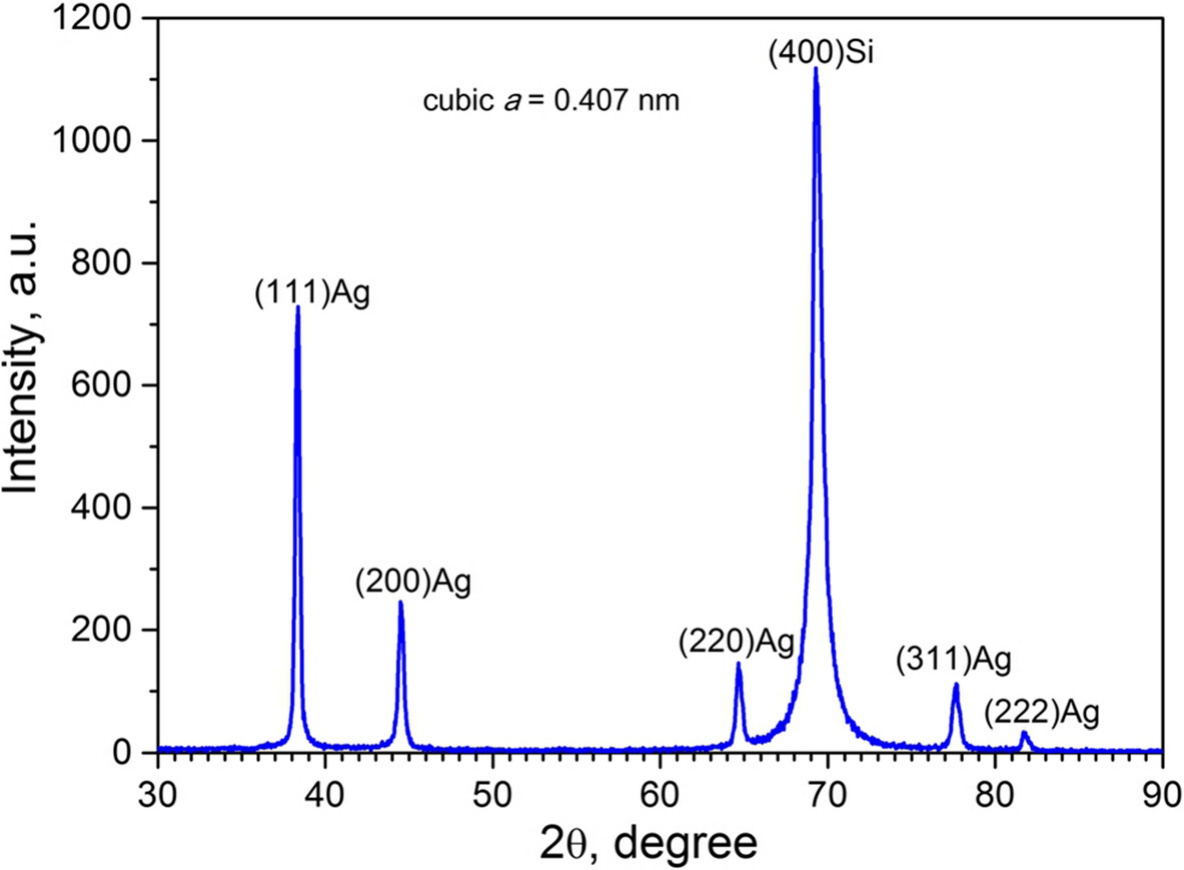
X-ray diffraction pattern of the Ag-coated PS. The Ag immersion deposition was carried out from the 1 mM AgNO_3_ solution for 120 min

Figure 1 presents the X-ray diffraction pattern of the PS sample after Ag immersion deposition from the 1 mM AgNO_3_ solution for 120 min. It can be seen that Ag deposit on the PS sample has a polycrystalline nature because the pattern contains characteristic peaks, which give an evidence of the cubic cell Ag crystals with orientations (111), (200), (220), and (311). The same view of the XRD pattern was typical for all Ag-coated PS samples distinguishing just in intensity of the characteristic peaks, which depended on the AgNO_3_ concentration and immersion time.

Using SEM analysis we revealed that in the regimes of the immersion, Ag deposit covers the PS top surface as the layer of particles. On the other hand, the inner surface of pore walls remains almost free of Ag NPs. We suggest that diffusion limitations in the pore channels prevent Ag^+^ ions penetration and deposition inside porous layer.

As it was mentioned in the “Background” section, the morphology of Ag coating on the PS surface strongly depends on the silver nitrate concentration and on the immersion deposition time. Aiming to explore regularities of the Ag nanostructures growth on PS and their correlation with an enhancement of the SERS signal, we prepared the Ag/PS samples using different combinations of these two parameters. Below three cases of the Ag deposit morphology resulted from the deposition in different solutions are considered: (i) 1 mM; (ii) 3 mM as a medium point within minimum and maximum concentration, and (iii) 10 mM.

### Morphology of the Ag/PS Samples Formed in the 1 mM AgNO_3_ Solution

**Fig. 2:**
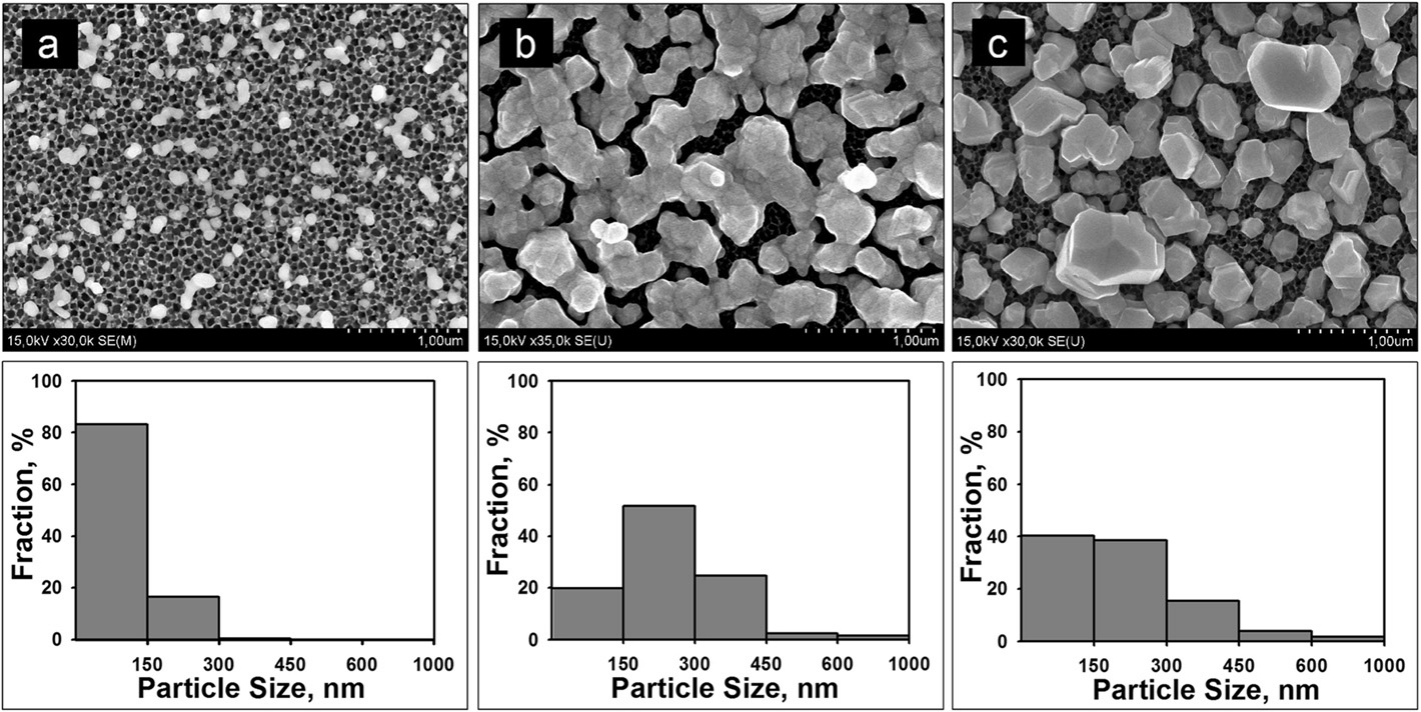
SEM top views of the PS samples after the immersion into 1 mM AgNO_3_ solution. Ag was deposited for **a** 15, **b** 120, and **c** 180 min. Below corresponding histograms of Ag NPs size distribution are presented. The histograms are normalized with respect to the total number of Ag NPs

Figure 2 shows SEM images of the top of the PS samples after immersion in 1 mM AgNO_3_ solution, which demonstrates typical morphology changes of the Ag coating during deposition process. Below the SEM images, corresponding size distribution histograms of Ag NPs are presented. As seen from Fig. 2, at the initial stage of deposition, the PS surface was slightly covered with the separated Ag NPs (islands) with diameters of a few to several tens of nanometers (Fig. 2a). The distance between Ag NPs varies from 150 to 500 nm. Further deposition of Ag led to the multiplication of the particles and increase of their dimensions resulted in the formation of Ag agglomerates. Most of Ag agglomerates are connected or located extremely close to each other (Fig. 2b) bearing the structure similar to a quasi-continuous film. We suggest that the Ag coating grew according to the Volmer–Weber mechanism [43] because formation of the Ag film followed by the growth of the initial Ag islands (Fig. 2a, b). Prolongation of the immersion deposition time up to 180 min caused the coalescence of the Ag agglomerates into large particles of an average diameter varied in the range of 400–800 nm (Fig. 2c). The coalescence takes a place due to an increase of the mutual attraction between Ag atoms during continuous deposition. This effect is typical for the Volmer–Weber mechanism as well. At the same time, disconnection of the Ag agglomerates at the coalescence process allowed reopening the PS surface for a growth of new Ag NPs. This effect is in correspondence with the model of the immersion deposition of metals on PS described earlier [32]. The analysis of the histograms reveals that size of Ag NPs for all the samples is favorable for the SERS effect (10–150 nm). Additionally, since the SERS activity is also affected by the mutual position of Ag NPs, i.e., the distance between them, the greatest intensity of the Raman signal is expected for the tightly packed but not connected Ag NPs. In our case, the distance between Ag NPs periodically varies depending on the deposition time. Based on this fact, one can suppose that the quasi-continuous Ag film (Fig. 2b) will demonstrate the maximum SERS performance, because the closely packed Ag agglomerates promote an abundance of vacancies for so-called hot spots, i.e., the scattering cross section of the analyte molecules should drastically increase.

### Morphology of the Ag/PS Samples Formed in the 3 and 10 mM AgNO_3_ Solutions

The similar mechanism of the Ag immersion deposition on PS was observed for 3 and 10 mM AgNO_3_ concentrations. However, the duration of each deposition stage went into decline upon the increase of the Ag^+^ ions number.

**Fig. 3:**
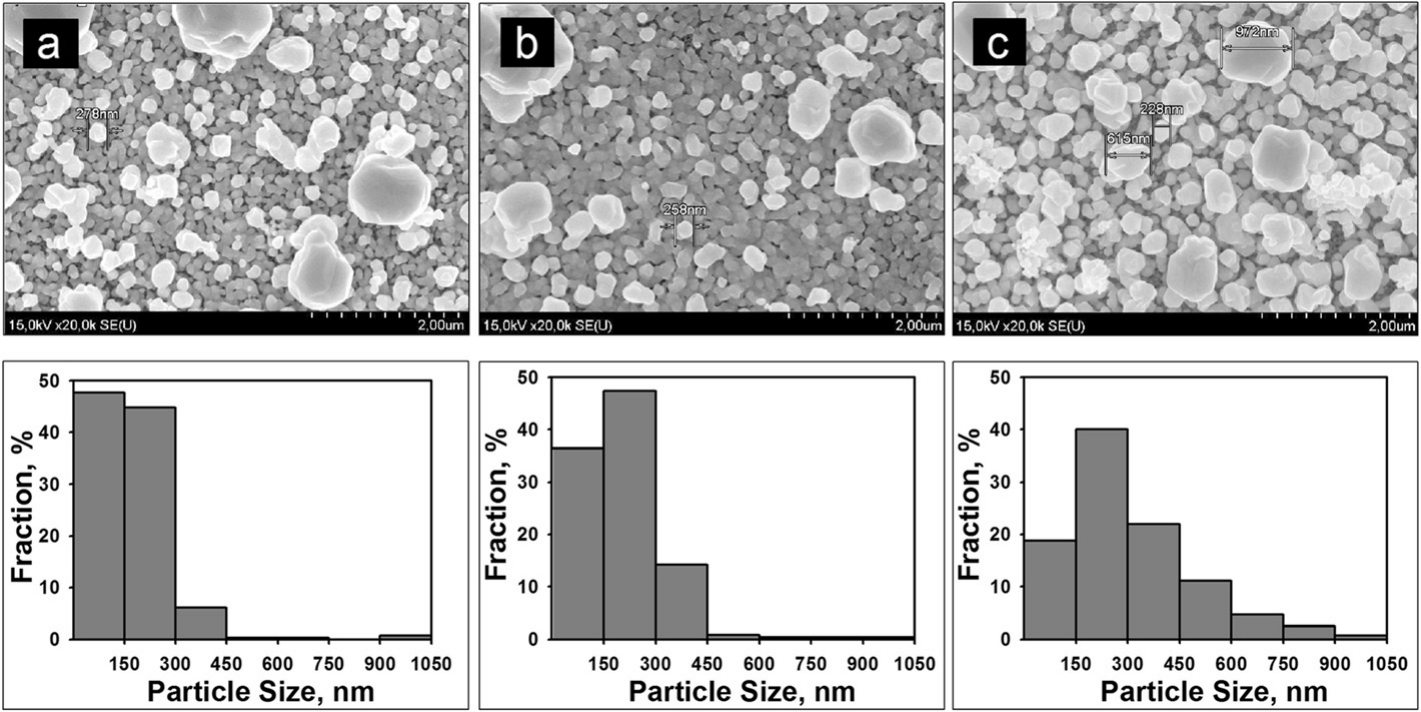
SEM top views of the PS samples after the immersion into 3 mM AgNO_3_ solution. Ag was deposited for **a** 15, **b** 120, and **c** 180 min. Below corresponding histograms of Ag NPs size distribution are presented. The histograms are normalized with respect to the total number of Ag NPs

**Fig. 4:**
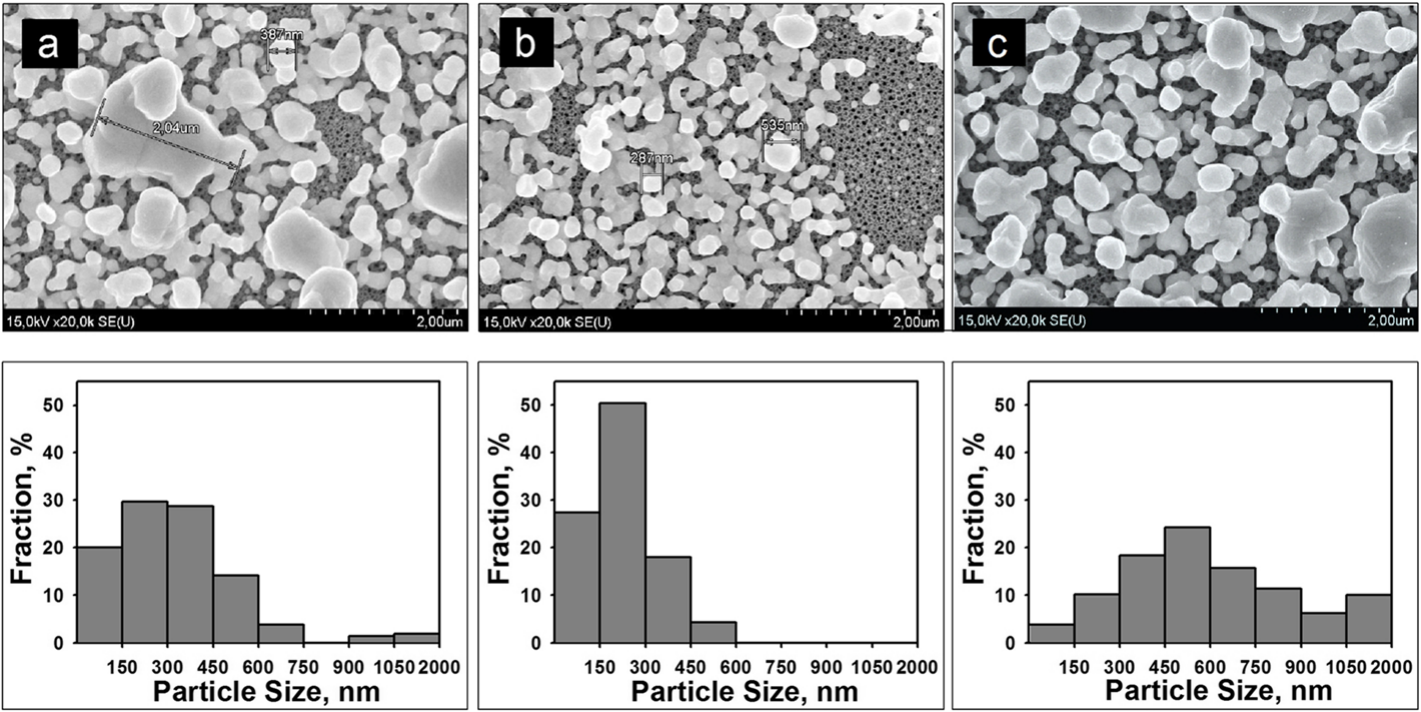
SEM top views of the PS samples after the immersion into 10 mM AgNO_3_ solution. Ag was deposited for **a** 15, **b** 120, and **c** 180 min. Below corresponding histograms of Ag NPs size distribution are presented. The histograms are normalized with respect to the total number of Ag NPs

Figures 3 and 4 show SEM images of the top of PS sample views after immersion in the 3 and 10 mM AgNO_3_ solutions, respectively. Corresponding size distribution histograms of Ag NPs are presented below each SEM image. In comparison with the Ag coating formed in 1 mM AgNO_3_ solution, the structures presented on Fig. 3 are characterized by more dense arrangement of Ag NPs. It should promote a relatively high density of the SERS-active sites. At the same time, Ag NPs in the films deposited from the 10 mM AgNO_3_ solution are arranged not as close to each other (Fig. 4). Hence, these Ag/PS samples are not expected to be very effective in the Raman signal enhancement.

Summarizing, there are similar regularities of the Ag deposition for all AgNO_3_ concentrations consisting in the alternation of the following steps: the growth of the layer of the individual particles and the formation of the quasi-continuous film.

### Reflectance Spectra

**Fig. 5:**
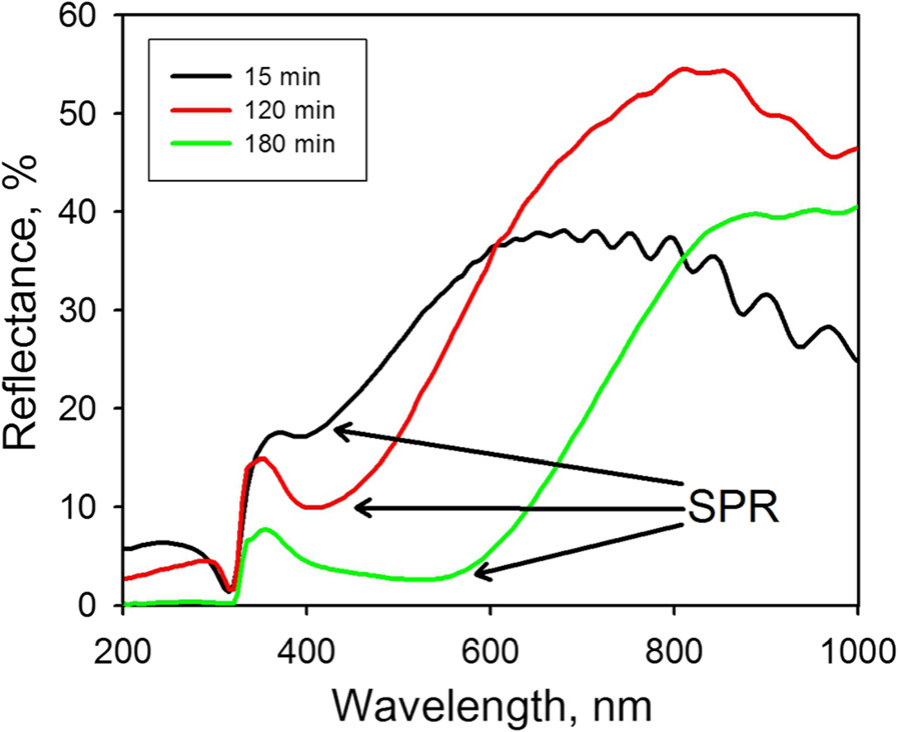
Reflectance spectra of the Ag/PS substrates. The substrates were formed by the immersion of PS in the 1 mM AgNO_3_ solution for different times

We characterized optical properties of the Ag/PS substrates in terms of SPR to reveal an appropriate excitation conditions for the SERS measurements. The position and intensity of SPR for the Ag nanostructures formed by the immersion deposition from the 1 mM AgNO_3_ solution for different periods are presented on Fig. 5. The spectra show marked bands corresponding to plasmon resonances related to absorption/scattering processes of the Ag coating. For the Ag/PS samples formed during 15 and 120 min, the SPR band is rather narrow and its position is located near 390–410 nm. For the sample fabricated by the longer deposition of Ag, the SPR band is quite wide and shifted to the 550-570 nm. The intensity of the SPR band of the second sample (120 min immersion deposition) is higher than that of other samples. Indirectly, it indicates a greater SERS activity of the corresponding sample jumping together with the conclusion made after the structural study of the samples. The observed behavior of the SPR bands can be explained by the gradual increase of the Ag NPs dimensions during their growth and an effect of the interparticle plasmonic coupling [45]. It should be noticed that such kind of the Ag nanostructures has a complex multilayer morphology where multipolar surface plasmon resonances can be realized.

### Dependence of the SERS Activity on the Ag Deposition Conditions

**Fig. 6:**
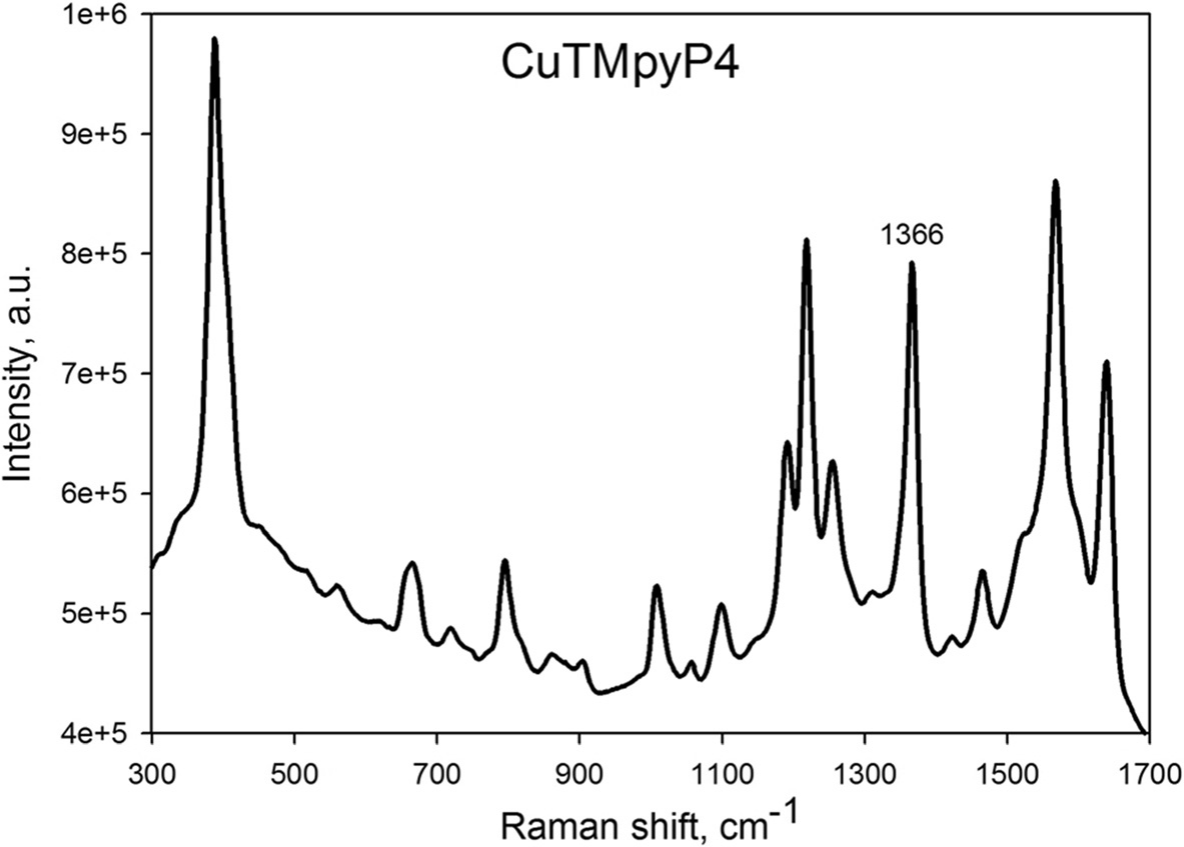
SERS spectrum of the analyte molecules adsorbed on the Ag/PS substrate. A 10^−6^ M CuTMpyP4 aqueous solution was used as analyte solution. The Ag/PS substrate was formed by the immersion of PS in the 1 mM AgNO_3_ solution for 120 min

A comparison of the Raman spectra intensity for the CuTMpyP4 molecules adsorbed on the PS and Ag/PS substrates revealed the SERS activity of the Ag-coated samples. The typical SERS spectrum of the CuTMpyP4 shown on Fig. 6 was observed for all Ag/PS substrates studied.

**Fig. 7:**
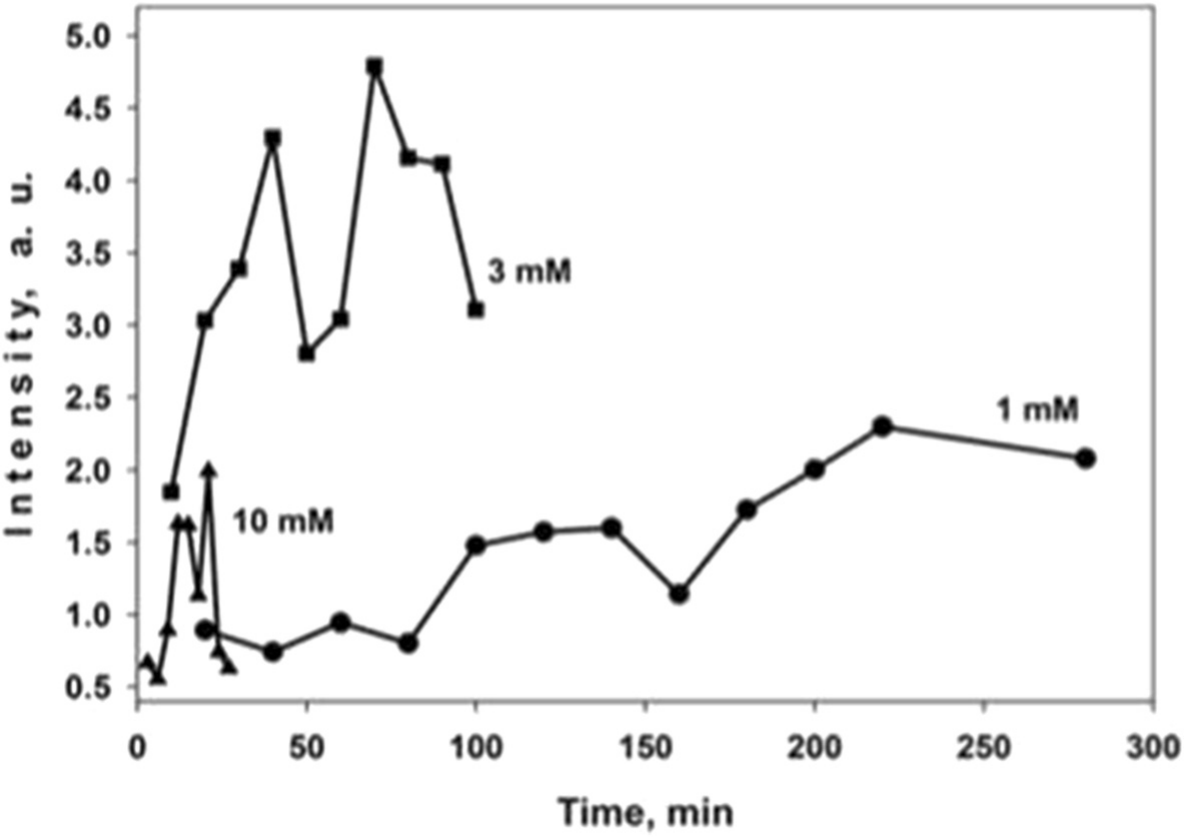
Dependence of SERS-signal intensity on the time of PS immersion in 1, 3 and 10 mM AgNO_3_ solutions. A 10^−6^ M CuTMpyP4 aqueous solution was used as analyte solution. The SERS-signal intensity of the 1366 cm^−1^ band in the SERS spectrum of 10^−6^ M CuTMpyP4 was estimated

Figure 7 shows the dependence of the 1366 cm^−1^ band intensity in the SERS spectrum of 10^−6^ M CuTMpyP4 on the immersion time for the samples of Ag/PS formed in 1, 3 and 10 mM AgNO_3_ solutions. Remarkable, the graphs have two peaks of the SERS-signal intensity. For example, in case of 1 mM concentration, they are located in the intervals of 100–140 and 200–270 min. The maxima of the Raman signal for the samples formed in 3 mM AgNO_3_ solution are related to the deposition during 40 and 70 min, while in the case of the 10 mM AgNO_3_ solution, they can be found at 12–15 and 21 min. It is reasonable to suggest that the observed behavior of the SERS-signal intensity is caused by the peculiarities of the Ag film growth described before. Therefore, to propose the origin of two maxima in SERS intensity, we put together the stages of the Ag deposition and the deposition periods resulted in the greatest SERS-signal intensity. The analysis of the Fig. 2 shows that in the Ag deposition beginning (15 min), there is a long distance between Ag NPs, which results in a low level of the Raman signal. The next morphological type of the quasi-continuous Ag film is favorable for the effective SERS due to closely packed Ag agglomerates, which promote the abundance of vacancies for the so-called hot spots, where the scattering cross section of the analyte’s molecules drastically increases. This structure matches with the first peak of the SERS-signal intensity. The following decrease of the SERS signal is related to the morphology where the Ag agglomerates partially disconnected and coalesced into the larger Ag structures (Fig. 2c). Finally, the new sub-layer of the small Ag NPs grew on the newly opened PS surface giving rise to the second maximum in the SERS-signal intensity.

The same correlation between the Ag-coating morphology and SERS-signal intensity can be found for the samples fabricated in the 3 and 10 mM AgNO_3_ solutions.

### Effective Time Parameter

Based on the performed analysis of the regularities of the Ag nanostructures growth via the Volmer–Weber mechanism and the SERS-signal intensity dependence on the Ag immersion time, we have proposed a universal parameter crucial for the choice of the optimal formation conditions of SERS-active substrates. This parameter was called an effective time (ET) and calculated as a product of the AgNO_3_ concentration ([AgNO_3_]) by the Ag immersion deposition time (*t*_imm_):

$$ \mathrm{E}\mathrm{T}={t}_{\mathrm{imm}}\cdot \left[{\mathrm{AgNO}}_3\right] $$

**Fig. 8:**
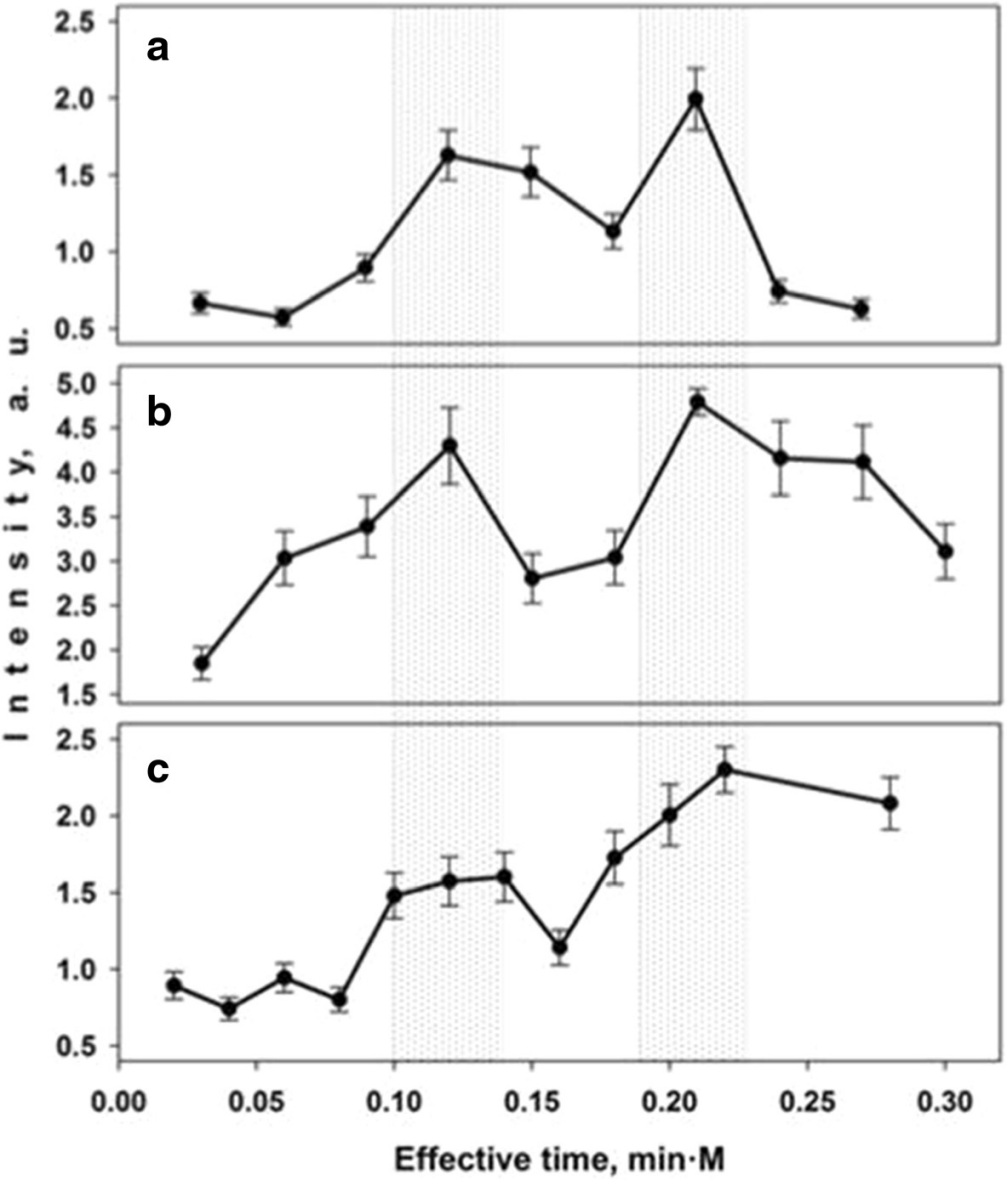
Dependence of SERS-signal intensity on effective time for **a** 1, **b** 3 and **c** 10 mM AgNO_3_ solutions. A 10^−6^ M CuTMpyP4 aqueous solution was used as analyte solution. The SERS-signal intensity of the 1366 cm^−1^ band in the SERS spectrum of 10^−6^ M CuTMpyP4 was estimated

Figure 8 demonstrates the dependence of the SERS-signal intensity on the effective time. For example, for the 1 mM AgNO_3_ concentration and 100 min immersion time ET = 0.1. In fact, two distinct maxima of the SERS-signal intensity are observed for three types of the Ag/PS substrates formed at the same effective times. Taking into account the efforts in the area of a technology development of highly effective SERS-active substrates with reproducible properties, we believe that the effective time is very useful for a choice of the conditions of the Ag immersion deposition on meso-PS.

### Detection Limit

**Fig. 9:**
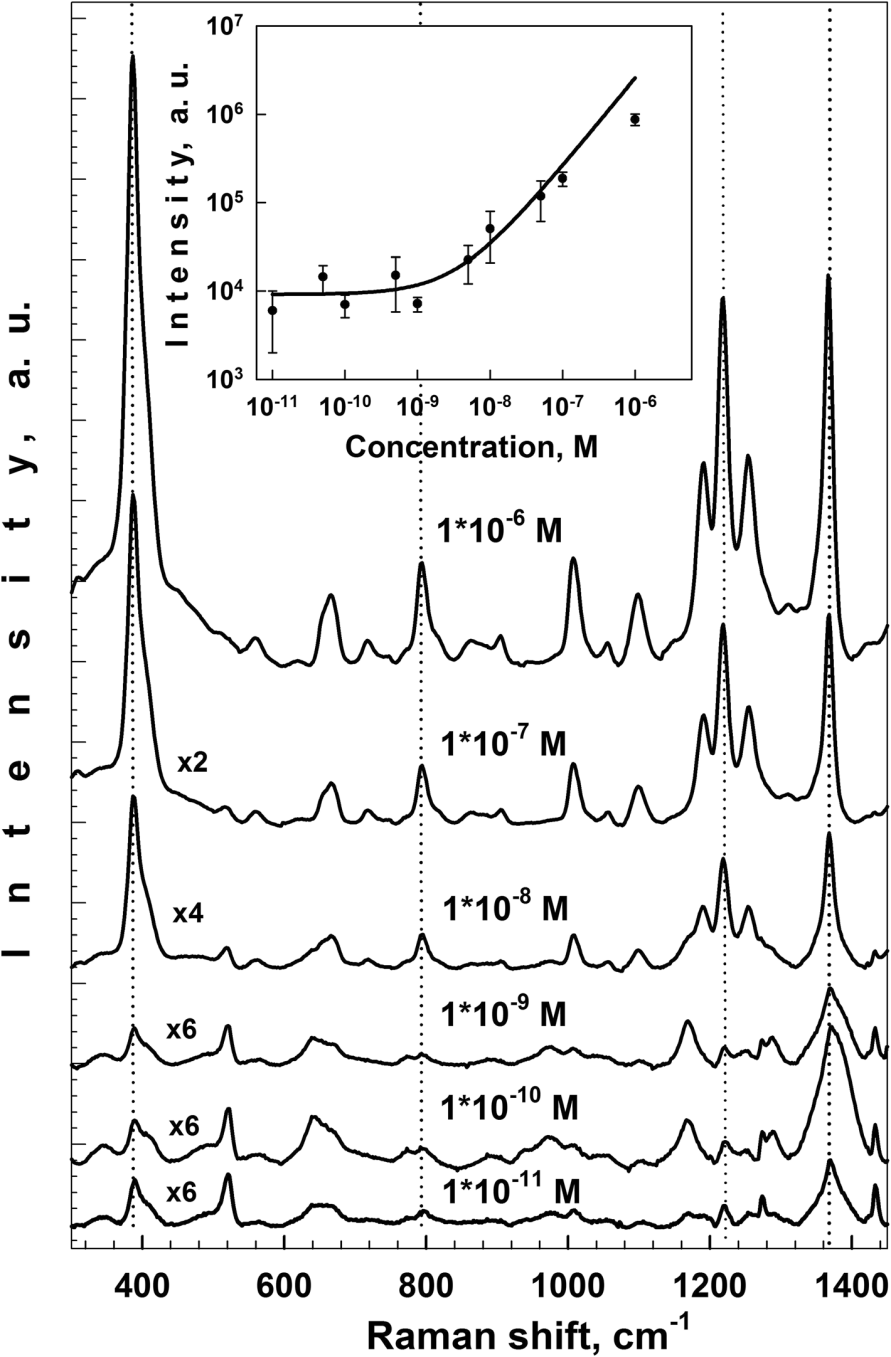
SERS spectra and detection limit of the CuTMpyP4 molecules adsorbed on the Ag/PS substrates. 10^−6^–10^−11^ M CuTMpyP4 aqueous solutions were used as analyte solutions. The Ag/PS substrates were formed by the immersion deposition of silver on PS from 3 mM AgNO_3_ solution for 40 min

One of the most adequate parameters traditionally used to characterize the SERS-active substrates is a detection limit. This parameter means a minimal concentration of analyte, which can be detected with the SERS-active substrate. In the previous section, we have demonstrated that the AgNO_3_ concentration does not affect the order of magnitude of the SERS-signal intensity (Fig. 7). Nevertheless, the SERS-signal intensity provided by the samples formed in the 3 mM AgNO_3_ solution is almost two times more than that of the substrates fabricated in the 1 and 10 mM AgNO_3_ solutions. That is why we evaluated the detection limit using the substrates prepared in the 3 mM AgNO_3_ solution for 40 min, because exactly this immersion time provides the most intensive SERS signal. Figure 9 shows SERS spectra and the detection limit of the CuTMpyP4 molecules adsorbed on the Ag/PS substrates from the solutions with different concentrations (10^−6^–10^−11^ M). As seen from Fig. 9, the detection limit of the Ag/PS substrates formed by the immersion deposition of silver for 40 min reaches 10^−11^ M.

## Conclusions

Plasmonic Ag nanostructures have been formed on the meso-PS by the immersion plating procedure. Morphological and optical properties of the fabricated Ag films were characterized by XRD, SEM, and UV–Vis reflectance spectroscopy. Raman enhancement has been evaluated using water-soluble cationic porphyrin CuTMPyP4. The maxima of the SERS-signal intensity were observed for the samples obtained by the PS immersion in the 3 mM AgNO_3_ aqueous solution with ethanol additive for 40 and 70 min. The detection limit for the CuTMPyP4 molecules adsorbed on Ag/PS surface was evaluated as 10^−11^ M. The comparative analysis of the data on the morphology and SERS activity of the Ag/PS substrates allows concluding that the optimal Ag films providing the maximal Raman enhancement for the same kind of the PS substrate can be formed taking into account a specific parameter called effective time*.* This parameter is a product of the AgNO_3_ concentration by the Ag immersion deposition time. The results obtained for the concentration/time relation are useful upon choice of the immersion reaction conditions for preparation SERS-active substrates based on mesoporous silicon.

## Abbreviations

NPs: nanoparticles
PS: porous silicon
SEM: scanning electron microscopy
SERS: surface-enhanced Raman scattering
SPR: surface plasmon resonance
XRD: X-ray diffraction
